# 57 A Comparison of Burn Rehabilitation Therapist’s Clinical Assessment vs Radiologic Detection of Heterotopic Ossification Formation

**DOI:** 10.1093/jbcr/iraf019.057

**Published:** 2025-04-01

**Authors:** Andria Martinez, Anjali Raju, Renee Warthman, Claudia Islas, Karen Richey, Derek Murray, Kevin Foster

**Affiliations:** Diane & Bruce Halle Arizona Burn Center; Arizona State University; Diane & Bruce Halle Arizona Burn Center; Diane & Bruce Halle Arizona Burn Center; Diane & Bruce Halle Arizona Burn Center; Diane & Bruce Halle Arizona Burn Center; Diane & Bruce Halle Arizona Burn Center

## Abstract

**Introduction:**

Heterotopic Ossification (HO), the development of abnormal bone in soft tissue that typically surrounds a joint can be severely debilitating. HO symptoms include intolerable pain, decreased range of motion (ROM), and change in joint end feel. Complications such as joint fusion impact quality of life, function, and total recovery. Therapists work closely with patients and may become aware of symptoms before a formal imaging diagnosis is made. The purpose of this study was to identify the difference in time from clinical assessment to imaging conformation in HO formation at our center.

**Methods:**

This was a retrospective study of patients, over a 10-year period, who developed HO to the elbow during their initial hospitalization. Data collection included basic demographic, injury and hospital data. Data specific to study objectives included diagnostic imaging and therapy assessments. ROM was categorized into mild, moderate, severe, and within normal limits (WNL). Symptoms that lead to suspicion of HO presence were categorized into pain, ROM, and end feel. Descriptive statistics were calculated.

**Results:**

There was a total of 48 study subjects. The majority were male (n=42, 88%) flame was the most common mechanism of injury (n=43, 90%), µ TBSA 51% (range 22-95%), µ ventilator days, 77 and µ length of stay was 110 days. Emergence of symptoms were categorized into ROM (n=24), pain (n=15), and joint end feel (n=9). The mean difference of suspected HO to imaging conformation was 29.9 days (range 0- 167, ± 42.3). The mean number of days from suspected HO to start of work up was 7 days (range 0-93, ± 17.7). Average number of images prior to conformation of HO was 1.02 (range 0-4, ±1.02). Following suspicion of HO, goniometry was obtained to the elbow on average 8.9 (range 0-58, ±13.3) ROM of elbow was also collected at or near discharge and categorized into WNL (n=7), mild (n=9), moderate (n=8), and severe (n=23).

**Conclusions:**

Our study suggests that clinical assessment by burn rehabilitation therapists demonstrates increased sensitivity in detecting heterotopic ossification formation when compared to diagnostic imaging.

**Applicability of Research to Practice:**

Clinical assessment focusing on progressive decreasing ROM, intolerable increase in pain, as well as firm to hard humeroradial & humeroulnar end feel, may assist the transdisciplinary team to make informed decisions for the patient and their care plan.

**Funding for the Study:**

N/A

